# Bloodmeal host identification with inferences to feeding habits of a fish-fed mosquito, *Aedes baisasi*

**DOI:** 10.1038/s41598-019-40509-6

**Published:** 2019-03-08

**Authors:** Takashi Miyake, Natsuki Aihara, Ken Maeda, Chuya Shinzato, Ryo Koyanagi, Hirozumi Kobayashi, Kazunori Yamahira

**Affiliations:** 1 Faculty of Education, Gifu University, Gifu, 501-1193 Japan; 2 Okinawa Institute of Science and Technology Graduate University (OIST), Onna, Okinawa 904-0495 Japan; 3 Atmosphere and Ocean Research Institute, The University of Tokyo, Chiba, 277-8564 Japan; 4 Graduate School of Engineering and Science, University of the Ryukyus, Nishihara, Okinawa 903-0213 Japan; 5 Tropical Biosphere Research Center, University of the Ryukyus, Nishihara, Okinawa 903-0213 Japan

## Abstract

The mosquito, *Aedes baisasi*, which inhabits brackish mangrove swamps, is known to feed on fish. However, its host assemblage has not been investigated at the species level. We amplified and sequenced the cytochrome oxidase subunit I barcoding regions as well as some other regions from blood-fed females to identify host assemblages in the natural populations from four islands in the Ryukyu Archipelago. Hosts were identified from 230 females. We identified 15 host fish species belonging to eight families and four orders. Contrary to expectations from previous observations, mudskippers were detected from only 3% of blood-engorged females. The dominant host was a four-eyed sleeper, *Bostrychus sinensis* (Butidae, Gobiiformes), in Iriomote-jima Island (61%), while it was a snake eel, *Pisodonophis boro* (Ophichthidae, Anguilliformes), in Amami-oshima and Okinawa-jima islands (78% and 79%, respectively). Most of the identified hosts were known as air-breathing or amphibious fishes that inhabit mangroves or lagoons. Our results suggest that *A. baisasi* females locate the bloodmeal hosts within the mangrove forests and sometimes in the adjacent lagoons and land on the surface of available amphibious or other air-breathing fishes exposed in the air to feed on their blood.

## Introduction

For most mosquito species, blood proteins are essential nutrients for egg production and thus, for reproductive fitness^1^. Therefore mosquito species have evolved to utilize the vertebrates as hosts (bloodmeals)^2^. When a number of possible hosts exist in their habitats, host preferences may develop. This may be the case, especially if blood quality affects reproductive output^1^.

The range of host vertebrates and host preferences has been investigated intensively for species that feed on humans^1,2^ as they can be vectors of pathogens that cause a variety of infectious diseases, including malaria, dengue fever, Japanese encephalitis, West Nile fever, etc. In the 1980s to 1990s, the enzyme-linked immunosorbent assay (ELISA) was mainly used for host identification^3,4^. However, this technique requires anti-sera preparation, for which the researchers need to identify candidate hosts before species-level identification. Otherwise, they can first apply anti-sera with broad activity (e.g., anti-bird and anti-mammal), but additional steps are needed for identification at the species level^5^.

DNA sequencing-based identification has been popular for the last decade. Development of vertebrate-specific primers for the regions frequently used for phylogeny analysis represented a breakthrough using this approach (e.g., mitochondrial 12S rRNA and 16S rRNA^6^, cytochrome b^4^ and cytochrome oxidase subunit I^4,7^). Studies on bloodmeal hosts from human-fed mosquito species using this approach have identified the broad range of host taxa including mammals, birds, reptiles^8–10^ and amphibians^4,9^ to the species level.

Some mosquito species, such as *Culex peccator*, *C. erraticus, Aedes albopictus, A. togoi*, and several *Uranotaenia* spp., are known to feed mainly on ectothermic hosts^9,11,12^. However, there are few species reported to feed on fish. Slooff and Marks^13^ observed *A. longiforceps* feeding on a mudskipper, *Periophthalmus musgravei*, in the Solomon Islands. Okudo *et al*.^14^ observed *Aedes baisasi* feeding on another mudskipper, *Pe. argentilineatus* in a cage and confirmed by ELISA using anti-fish antibodies that mosquitoes collected in the field had fed on fish. These authors suggested that mudskippers should be most readily accessible hosts because they are very common and are out of water for long periods. Tamashiro *et al*.^9^ further investigated the host range of wild *A. baisasi* using DNA sequencing of the 16S rRNA region and found DNA sequences of fish origin in 94% of blood-engorged *A. baisasi* females (the remaining 6% had fed on frogs). BLAST searches resulted in either a goby (Gobiiformes) or a snake eel (Anguilliformes) with relatively low similarities (91–92% and 89–95%, respectively) due to a deficiency of 16S rRNA data of potential candidates in the NCBI database, and they could not identify the host species.

**Figure 1:**
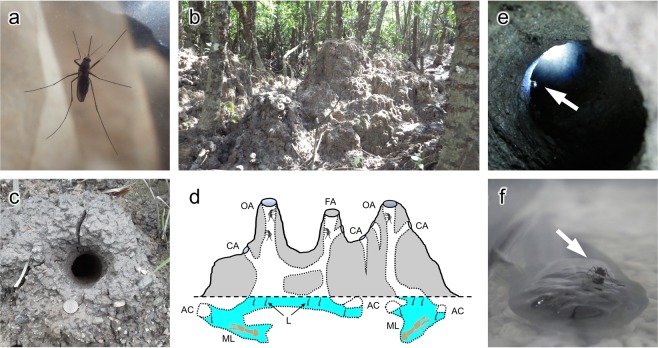
The life-history of *Aedes baisasi*. (**a**) An adult female. (**b**) Mounds made by a mud lobster *Thalassina anomala* in a mangrove forest. (**c**) Aperture of a burrow in the mound. The coin is 22.6 mm in diameter. (**d**) Hypothesized structure of a mound, modified with permission from Miyagi & Toma^57^. Apertures of lobster burrows are basically open (OA), but are sometimes filled with mud (FA). Apertures of burrows made by crabs on the mounds (CA) are often connected to those made by the lobster. The lower part of the burrow is filled with brackish water, where *A. baisasi* larvae (L) live. Mud lobsters (ML) usually stay in brackish water and in an air chamber (AC) in the burrow. (**e**) An adult resting on the wall of the burrow (arrow). (**f**) A female feeding on the exposed surface of *Bostrychus sinensis* (arrow) in an enclosed cage during a preliminary experiment. Photo credits: Takashi Miyake.

*Aedes baisasi*^15^ (Fig. 1a) inhabits burrows made by a mud lobster *Thalassina anomala* or land crabs such as *Cardisoma carnifex*, *Discoplax hirtipes*, *and Episesarma lafondii*, which are often found in the upper intertidal zone of mangrove forests in the Ryukyu Archipelago^14,16^ (Fig. 1). Larvae grow in brackish water (salinity 0–31) within burrows^16^. Adults are nocturnal; they rest in the upper parts of the holes in the daytime and fly out at night. Physiological states of females collected in the burrows suggest that adult females have a short flight range and that their mating and feeding activities are restricted to mangroves close to the burrows^16^. Therefore, it is expected that most candidate hosts also inhabit mangroves.

Accordingly, we determined mitochondrial genome sequences of 11 species from the orders Gobiiformes and Anguilliformes that are expected to appear near mangroves on Okinawa-jima and Iriomote-jima to enhance the reference database (Supplementary Table S1). We also determined 15,400 bp of the mitochondrial DNA sequence of a snake eel *Pisodonophis boro* (Anguilliformes; An2 in Table S1). Although we could not complete the entire circular mitochondrial genome, we used it as a reference, as it contains sequences of all regions used for host identification (cytochrome oxidase subunit I, cytochrome b, 12S rRNA, and 16S rRNA). In this study, specimens of the mosquito, *A. baisasi*, were collected from four islands in the Ryukyu Archipelago. With the enhanced database, we identified their hosts to the species level. In addition to the 16S r RNA, used in Tamashiro *et al*.^9^, we used three regions; (1) cytochrome oxidase subunit I, since data for this region has become increasingly available due to its use in ‘DNA barcoding’^17^, (2) cytochrome b, as this region has been used for phylogenetic analysis in the order Gobiiformes^18,19^, and (3) a hypervariable region of the 12S rRNA gene using the MiFish primer set developed for identification of fish species^20^. We demonstrate that *A. baisasi* uses a variety of host fishes and that host usage patterns vary among islands of the Ryukyu Archipelago. We discuss whether the broad host usage of *A. baisasi* and its inter-island variation reflect host preference.

## Results

### Host identification

We collected 758 adult females of *A. baisasi* from the four islands. Proportions of blood-engorged individuals among the females on each island ranged from 27.3 to 75.0% (Table 1). The highest proportion (75.0%) may have been due to small sample size (N = 8). We performed PCR amplification for 263 blood-engorged females and successfully amplified host DNA for 87.8% of them (Table 1).

**Table 1:** Numbers of specimens of *Aedes baisasi* collected, their status, and success rate of PCR amplification from their bloodmeals.

Population	Year	Male	Female			PCR		
			Blood-present	Blood-absent	% Blood-present	Female no.	Succeeded	% success
Iriomote-jima-1	2015	NA	52	83	38.5	52	50	96.2
Iriomote-jima-2	2015	NA	19	28	40.4	19	19	100.0
	2016	83	58	43	57.4	40	28	70.0
Ishigaki-jima	2015	NA	6	2	75.0	6	6	100.0
Okinawa-jima	2016	27	21	56	27.3	21	20	95.2
	2017	372	70	177	28.3	70	61	87.1
Amami-oshima	2017	129	60	83	42.0	55	47	85.5
Total			286	472	37.7	231	263	87.8

Of the COI sequences from 211 samples for which we conducted BLASTn searches against the GenBank database, only those from 96 samples achieved ≥99% similarity (Supplementary Table S2). Samples with 92–94% similarity to four-eyed sleeper *Bostrychus sinensis* (Butidae, Gobiiformes) sequences in the GenBank database, reached ≥99% similarity with *B. sinensis* in our supplementary database. All samples that resulted in ≥99% similarity to a conger eel *Uroconger lepturus* (Congridae, Anguilliformes) in the GenBank achieved ≥99% similarity to a snake eel *Pisodonophis boro* (Ophichthidae, Anguilliformes) in our database. We confirmed that BLASTn searches for the 12S rRNA sequences from some of these samples against the GenBank database resulted in same-species identification (*Pi. boro*) with ≥98% similarity (AMA9 and AMA10, for example, Supplementary Table S2). Accordingly, we concluded that *Pi. boro* was the correct host. We found considerable discrepancies among the sequences of both COI and 12S rRNA of a spaghetti eel, *Moringua microchir* (Moringuidae, Anguilliformes) within the GenBank database. Many of the 12S rRNA sequences from our samples matched one of them (accession no. LC020870) closely; thus, they were identified as *M. microchir*.

Combined with the GenBank database and ours (Supplementary Table S1), we could identify host fish species with ≥99% similarity from all 230 female mosquitoes analyzed (Table 2, Supplementary Fig. S1), but for one sample, intermingled DNA sequences were obtained for both COI and cytochrome b regions, which we considered as originated from two different blood sources (Supplementary Table S2), most likely from *Pi. boro* and *B. sinensis*. This sample was excluded from further analysis.

**Table 2:** Numbers of host species identified from abdomens of blood-engorged mosquitoes.

Order	Family	Species	Islands			
			Amami-oshima	Okinawa-jima	Ishigaki-jima	Iriomote-jima
Anguilliformes	Moringuidae	*Moringua microchir*	0	4	3	17
	Muraenidae	*Gymnothorax pictus*	0	0	0	1
		*Uropterygius concolor*	0	0	0	1
	Ophichthidae	*Pisodonophis boro*	36	64	0	5
Gobiiformes	Butidae	*Bostrychus sinensis*	9	11	0	60
	Oxudercidae	*Mugilogobius* sp. ‘Izumi-haze'	1	0	0	0
		*Periophthalmus argentilineatus*	0	1	0	6
		*Trypauchenopsis intermedia*	0	1	0	0
	Gobiidae	*Myersina macrostoma*	0	0	0	1
Blenniiformes	Blenniidae	*Blenniella bilitonensis*	0	0	1	0
		*Entomacrodus striatus*	0	0	0	1
		*Istiblennius edentulus*	0	0	2	0
		*Salarias fasciatus*	0	0	0	2
		*Salarias luctuosus*	0	0	0	2
Tetraodontiformes	Balistidae	*Rhinecanthus verrucosus*	0	0	0	1
Total			46	81	6	97

### Host assemblage

**Figure 2:**
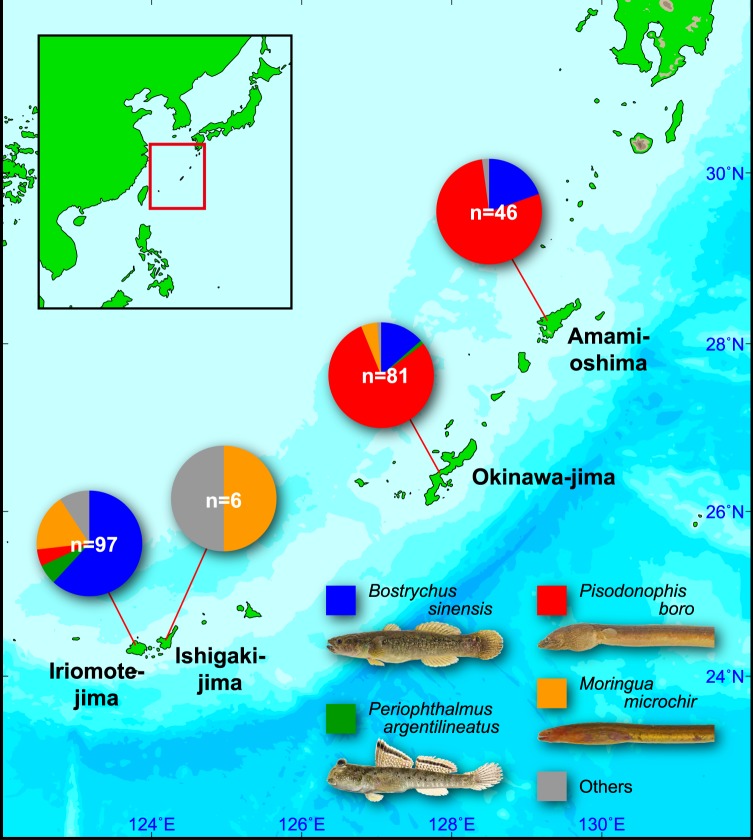
A map of study areas in the Ryukyu Archipelago, Japan and host assemblages of *Aedes baisasi* in each area. The main map was generated by GMT ver. 5.4.2 (http://gmt.soest.hawaii.edu/) and a template of the small scale map is from http://www.freemap.jp/. Photo credits: Ken Maeda.

Although we identified 15 host species from four islands, host assemblages were quite different among the islands, except for between Amami-oshima and Okinawa-jima (Fig. 2, Table 2). We identified bloodmeal hosts from 46 female mosquitoes in Amami-oshima, and only three host species were found, with *Pi. boro* as the most frequent host (78%). About 20% of all females fed on *B. sinensis*, and the blood from a goby *Mugilogobius* sp. ‘Izumi-haze’ was detected in one female. A similar pattern was observed in Okinawa-jima; five host species were found, again with *Pi. boro* as the most frequent host (79%) and *B. sinensis* as the second most frequent (14%). Some fed on *M. microchir* (5%) and the blood from a mudskipper, *Periophthalmus argentilineatus*, was detected in one female and from a bearded eel goby, *Trypauchenopsis intermedia*, in another.

On the other hand, we identified 11 host species from 97 female mosquitoes in Iriomote-jima. *Bostrychus sinensis* was the most frequent host (61%), followed by *M. microchir* (18%), *Pe. argentilineatus* (6%) and *Pi. boro* (5%). Some host species were found only in Iriomote-jima, but they are detected from only one or two female mosquitoes. These included three blennies *Entomacrodus striatus*, *Salarias fasciatus* and *S. luctuosus* (Blenniidae, Blenniiformes), a goby *Myersina macrostoma* (Gobiidae, Gobiiformes), two moray eels *Gymnothorax pictus* and *Uropterygius concolor* (Muraenidae, Anguilliformes), and a triggerfish *Rhinecanthus verrucosus* (Balistidae, Tetraodontiformes).

Although we collected only 6 blood-engorged females on Ishigaki-jima, host composition was quite different from the other islands; *M. microchir* was the most frequent (50%), followed by two blennies *Blenniella bilitonensis* and *Istiblennius edentulous*.

## Discussion

### Host identification based on DNA sequences

The DNA-sequence-based technique has brought great progress in bloodmeal host identification of mosquitoes and other arthropods (e.g., biting midges^21–23^ and ticks^7,24^). In our study, using a combination of some DNA regions used for phylogenetic studies and DNA barcoding, host identification for fish-fed *A. baisasi* was much improved. Tamashiro *et al*.^9^ aimed at revealing feeding habits of 35 mosquito species from 11 genera with broad spectra of potential host animals. They also used the mitochondrial 16S rRNA region for this species and this enabled identification at higher taxonomic levels (i.e., at the order level). In our study, we applied the COI region to host identification^4,7^ and also benefited from recently developed universal PCR primers, which were originally designed for metabarcoding environmental DNA from fishes^20^. This resulted in host identification at the species level. Therefore, this study was the first to demonstrate host assemblages of mosquito species that are parasitic upon fish in natural populations.

While the COI region is well established for DNA barcoding and the amount of available data has been increasing, many of our samples failed to surpass 99% similarity to any taxa in GenBank. There are several possible reasons for this. First, the GenBank database does not include sequence diversity among localities. For example, *B. sinensis* is widely distributed and regional differentiation may account for the low similarity (92–94%). Second, taxonomy has often not been clearly established and that makes it difficult to complete the database. Regardless, we developed a database including more species from more localities with reliable identification and the genomic data. The sequence database of candidate fishes from the sampling areas greatly improved the similarity, and the short fragment of 12S rRNA (using a “MiFish” primer set)^20^ helped to further resolve these problems (Supplementary Table S2).

### Feeding habits of *Aedes baisasi*

Host identification at the species level has some interesting implications. First, contrary to our expectations, based on the laboratory observation by Okudo *et al*.^14^, mudskippers were not the main source of bloodmeals in *A. baisasi*; DNA sequences that originated with a mudskipper *Periophthalmus argentilineatus* were detected in only 7 out of 229 (3%) host-identified females. Second, bloodmeal hosts fed frequently on fish species which inhabit mangrove swamps, such as *B. sinensis, Pi. boro* and *Pe. argentilineatus*^25,26^, confirming that feeding activities of *A. baisasi* are restricted to the mangroves^16^. Some other species documented less frequently included *Mugilogobius* sp*., Myersina macrostoma, Trypauchenopsis intermedia*, and *Uropterygius concolor*, which also inhabit mangroves^26–28^. Some species, such as *Entomacrodus striatus*, *Salarias fasciatus*, *Istiblennius edentulus*, and *Gymnothorax pictus* inhabit tidepools along rocky shore and/or coral reefs^29^, implying that *A. baisasi* may sometimes search for hosts out of mangrove forests and may visit nearby lagoons. Third, many bloodmeal hosts can remain out of water for prolonged periods. *Bostrychus sinensis* is known as a facultative air-breathing fish^30^, and can survive out of water for more than a day^31^. *Pisodonophis boro* also has air-breathing habits^32^. Some blennies are amphibious, e.g., *E. striatus*^33–35^, *I. edentulus*^35,36^ (but see Platt *et al*.^33^) and also *B. bilitonensis*, sometimes clings to rocks out of water^37^. The moray eel *G. pictus* also leaves water and wriggles across dry places^38^. Given the behavioral attributes of these fishes, it is likely that *A. baisasi* searches for and lands on fishes either when they leave the water or when parts of their bodies are exposed to the air.

Many of these air-breathing or amphibious fish are reported to be more active out of the water at night than during the day. Most amphibious blennies emerge from water mainly at night to avoid the risk of desiccation^39,40^. *Bostrychus sinensis* usually hides in hollows on mud, beneath rocks, or gaps in mangrove roots, and in caves during the daytime and emerges at night^41^. We have seen many individuals feeding in shallow waters at night, which may often expose themselves to the air. *Pisodonophis boro* is also active at night at shallows^42^, foraging mainly for sesarmid crabs^43^, and is also an accessible host for mosquitoes. These habits match the feeding activity of *A. baisasi*.

Our results, with support from other observations^14,16^, suggest that females rest in lobster holes during the daytime and leave at night to search for bloodmeal hosts within the mangroves and sometimes in adjacent lagoons. They locate and land on the surfaces of exposed amphibious or other air-breathing fishes and feed there, but are not attracted to humans or warm-blooded animals^14^. However, some ecological factors remain unclear regarding seasonal variation in host selection, the stage of fishes on which *A. baisasi* feed (e.g., juveniles, immatures or adults) and the cues *A. baisasi* uses to locate bloodmeal hosts. Seasonal variation is expected because fish communities vary seasonally in mangroves^25,42^. Tamashiro *et al*.^9^ found three *A. baisasi* females with blood from frogs. These were sampled on Iriomote-jima in May (Ichiro Miyagi and Takako Toma, Laboratory of Mosquito Systematics of Southeast Asia and South Pacific, personal communication). Olfaction is the likely cue for the mosquitoes to locate a host, which is implied by the fact that air-breathing fishes have special excretion systems in their skin^44–46^, but laboratory and/or field assays are needed to confirm this hypothesis.

### Host preference of *Aedes baisasi*

Host assemblages were quite different among the islands. This may reflect differences in abundance of available fishes among the islands, considering that *B. sinensis* is abundant on Iriomote-jima, but less on Okinawa-jima and Amami-oshima^41,47^.

On the other hand, it is puzzling that *A. baisasi* do not feed frequently on the mudskipper, *Pe. argentilineatus*, which is apparently the most accessible fish host in this region. *Periophthalmus argentilineatus* seems less active at night^48^, and we often saw it still staying out of water. The mudskipper may have some kind of mechanism that keeps off *A. baisasi*. It is also the puzzling that significant blood feeding from spaghetti eels, *Moringua microchir*, occurred on three of the four islands. Little information is available on the life history and ecology of moringuid eels, including *M. microchir*^29,49^*. Moringua microchir* is recognized as a rather rare species in Japan, and it has not reported from mangroves. Keith *et al*.^50^ reported that the juveniles inhabit estuaries and lower reaches of rivers, while adult females stay on shallow marine bottoms. However, frequent predation by mosquitoes implies that spaghetti eels are actually common around mangroves. Our data also suggest that *A. baisasi* may preferentially feed on it. Although *A. baisasi* chooses bloodmeal hosts according to their availability (abundance and nocturnal activity out of water), host preferences may exist.

The subgenus *Geoskusea*, to which *A. baisasi* belongs, includes 10 species, all of which also inhabit brackish water^51^. In addition to *A. baisasi* and *A. longiforceps*^13^, other species also probably use air-breathing fishes as bloodmeal hosts. Further studies of host identification in these species will help our understanding of the evolution in host preference and exploitation in niche adaptation.

## Methods

### Sampling

Adult mosquitoes were collected during the daytime (between 9:00 a.m. and 6:00 p.m.) in mangrove forests on four islands in the Ryukyu Archipelago, Japan (Fig. 2): Iriomote-jima Island (Iriomote-jima-1: Uehara, 24° 23′ N, 123° 49′ E, and Iriomote-jima-2: Komi, 24° 19′ N, 123° 54′ E) in November, 2015 and October, 2016, Ishigaki-jima Island (24° 25′ N, 124° 14′ E) in November, 2015, Okinawa-jima Island (24° 19′ N, 123° 54′ E) in October, 2016 and September, 2017, and Amami-oshima Island (28° 15′ N, 129° 24′ E) in September, 2017. Mosquitoes resting in crab or mangrove lobster burrows during the daytime^16^ were forced out by disturbing them with a twig and trapped with a sweep net. We found from preliminary surveys that it was more effective than using a handheld vacuum^16^. Collected mosquitoes were preserved in 99.5% ethanol and brought to the laboratory.

### Bloodmeal host identification

Each mosquito was individually examined to determine species, sex, and blood-feeding status under a microscope. For blood-engorged females, their abdomens were isolated using forceps. Genomic DNA was extracted from the abdomen either using a DNeasy Blood & Tissue Kit (Qiagen) or by the HotSHOT method^52^.

We conducted PCR to amplify either mitochondrial COI (cytochrome oxidase subunit I) with primers M13BC-FW (5′-TGT AAA ACG ACG GCC AGT HAA YCA YAA RGA YAT YGG NAC-3′) and COI_long (r) (5′-AAG AAT CAG AAT ARG TGT TG-3′) designed to amplify exclusively from vertebrate DNA^4,7^, or mitochondrial 12S rRNA region with M13F-attached MiFish-U-F (5′-GTA AAA CGA CGG CCA GGT CGG TAA AAC TCG TGC CAG C-3′) and MiFish-U-R (5′-CAT AGT GGG GTA TCT AAT CCC AGT TTG-3′)^20^. PCR was performed using KOD FX Neo DNA polymerase kit (Toyobo, Japan) in a 20 µL total volume: 5 µL of 2x Buffer, 0.3 µL of 10 µM each primer, 2 µL of 2 mM dNTPs, 0.2 µL of KOD FX Neo, 0.4 µl of template DNA and 1.8 µL of dH_2_O. PCR cycling conditions were as follows: initial denaturation at 94 °C for 2 min, then 35 cycles of 98 °C for 10 s, 48 °C for 30 s, and 68 °C for 1 min for COI, and 35 cycles of 98 °C for 10 s, 50 °C for 10 s, and 68 °C for 15 s for 12S rRNA. PCR products were visualized on 1.5% agarose gels to confirm amplification. Amplified products were purified using Exo-SAP-IT (USB corp., Cleveland, OH, USA). For both regions, sequence reactions were conducted with M13F primer (5′-GTA AAA CGA CGG CCA G-3′) using the BigDye Terminator v3.1 Cycle Sequencing Kit (Applied Biosytems). Sequencing products were analyzed on an ABI PRISM 3130 capillary DNA sequencer (Applied Biosystems).

All sequenced specimens were identified using BLASTn searches against the GenBank nucleotic acid sequence database (NCBI website, http://www.ncbi.nlm.nih.gov/BLAST/) and/or a similar search using BLAST+ ver. 2.3.0+ against the additional reference sequences stated below. The most similar fish species (≥99% sequence identity) based upon blood from engorged female mosquitoes was considered to be the parasitized host.

When we could not find any data that matched our query sequences at ≥99%, we conducted PCR using the same DNA polymerase kit for other regions with primers designed to amplify exclusively from vertebrate or fish DNA: the mitochondrial cytochrome B region with CytB(f)(5′-GAG GMC AAA TAT CMT TCT GAG G-3′) and CytB(r)(5′-TAG GGC VAG KAC TCC TCC TAG T-3′)^4^, or the mitochondrial 16S rDNA region with 16Sa-L (5′-CGC CTG TTT ACC AAA AAC ATC GCC T-3′) and 16Sb-H (5′-CCG GTC TGA ACT CAG ATC ACG T-3′)^53^. PCR cycling conditions were as follows: initial denaturation at 94 °C for 2 min, then 35 cycles of 98 °C for 10 s, 55 °C for 40 s, and 68 °C for 1 min for cytochrome B, and 35 cycles of 98 °C for 10 s, 50 °C for 40 s, and 68 °C for 1 min for 16S rDNA. Positive amplicons were sequenced with one of the primers used in PCR reactions. Identities of all sequenced specimens were determined as described above.

### Reference sequences of fishes

Total genomic DNA of 15 specimens belonging to six anguilliform species and six gobiiform species collected from Okinawa-jima and Iriomote-jima was extracted from the right pectoral fins (Gobiiformes) or muscle pieces (Anguilliformes) preserved in 99.5% ethanol, using a DNeasy Blood & Tissue Kit (Quiagen, Hilden, Germany) or a Maxwell RSC Blood DNA Kit (Promega, Fitchburg, Wisconsin, USA).

Whole genome shotgun sequencing libraries were prepared using a KAPA HyperPlus Kit, PCR-free (KAPA Biosystems, Wilmington, Massachusetts, USA). Extracted genomic DNA was enzymatically fragmented into pieces of 200–1000 bp. After repairing the protruding ends and A-tailing, sequencing adaptors were ligated onto both ends of the DNA fragments. Shotgun libraries were then sequenced on either an Illumina MiSeq sequencer (Illumina, San Diego, California, USA) with MiSeq V3 600 cycle kit (Illumina) or an Illumina HiSeq 2500 sequencer in Rapid Run mode version 2 using a HiSeq Rapid Cluster Kit v2-Paired-End (Illumina) and HiSeq Rapid SBS Kit v2 (Illumina) or an Illumina HiSeq 4000 sequencer with HiSeq 3000/4000 PE Cluster Kits and HiSeq 3000/4000 SBS kit (300 cycles, Illumina) following manufacturer instructions.

Sequencing data from each library were assembled with the IDBA_UD assembler version 1.1.1^54^ with different kmer lengths (60, 80, 100). Identification of complete mitochondrial genomes from assembled contigs was performed by (1) comparing them with the complete *Stiphodon alcedo* mitochondrial genome (accession: AB613000.1) (BLASTN e-value B 1e-100), and by (2) confirming that 100 bp of both head and tail DNA sequences of a contig were identical, indicating that the sequence was circular. Complete mitochondrial genomes were aligned using MAFFT v7.244^55^ and all positions with gaps were removed using trimAl^56^. All sequenced raw data are available in the DDBJ Sequence Read Archive under BioProject accession number PRJDB5763. Assembled mitochondrial genome sequences with gene annotations are available in the DDBJ database under accession numbers: AP019348–AP019362. Accession numbers for each individual are shown in Supplementary Table S1.

Procedures used to handle fish specimens in this study were approved by the Animal Care and Use Committees of both Okinawa Institute of Science and technology Graduate University and Gifu University. All experiments and samplings were performed in accordance with relevant guidelines and regulations of the committees.

## Supplementary information

Dataset 1

Dataset 2
