# Supplementary material for: Bloodmeal host identification with inferences to feeding habits of a fish-fed mosquito, Aedes baisasi
Source: Sci Rep. 2019 Mar 8;9:4002. doi: 10.1038/s41598-019-40509-6 (PMC6408532; doi:10.1038/s41598-019-40509-6)
Supplement: Supplementary file 2 — Dataset 2 [file 41598_2019_40509_MOESM2_ESM.pdf]

Scientific reports Supplementary Information Figure S1

Bloodmeal host identification with inferences to feeding habits of a fish-fed mosquito, *Aedes baisasi*

Takashi Miyake*, Natsuki Aihara, Ken Maeda, Chuya Shinzato, Ryo Koyanagi, Hirozumi Kobayashi & Kazunori Yamahira

*corresponding author, tmiyascb@gifu-u.ac.jp

Supplementary information Figure S1. Pictures of bloodmeal-host species of the mosquito *Aedes baisasi* in the Ryukyu Archipelago. (a) *Moringua microchir*; (b) *Pisodonophis boro*; (c) *Uropterygius concolor*; (d) *Gymnothorax pictus*; (e) *Periophthalmus argentilineatus*; (f) *Trypauchenopsis intermedia*; (g) *Mugilogobius* sp. ‘Izumi-haze’; (h) *Bostrychus sinensis*; (i) *Myersina macrostoma*; (j) *Entomacrodus striatus*; (k) *Istiblennius edentulus*; (l) *Salarias luctuosus*; (m) *Salarias fasciatus*; (n) *Blenniella bilitonensis*; (o) *Rhinecanthus verrucosus*; photo credits: a–c and e–n (Ken Maeda); d and o (Katsunori Tachihara).

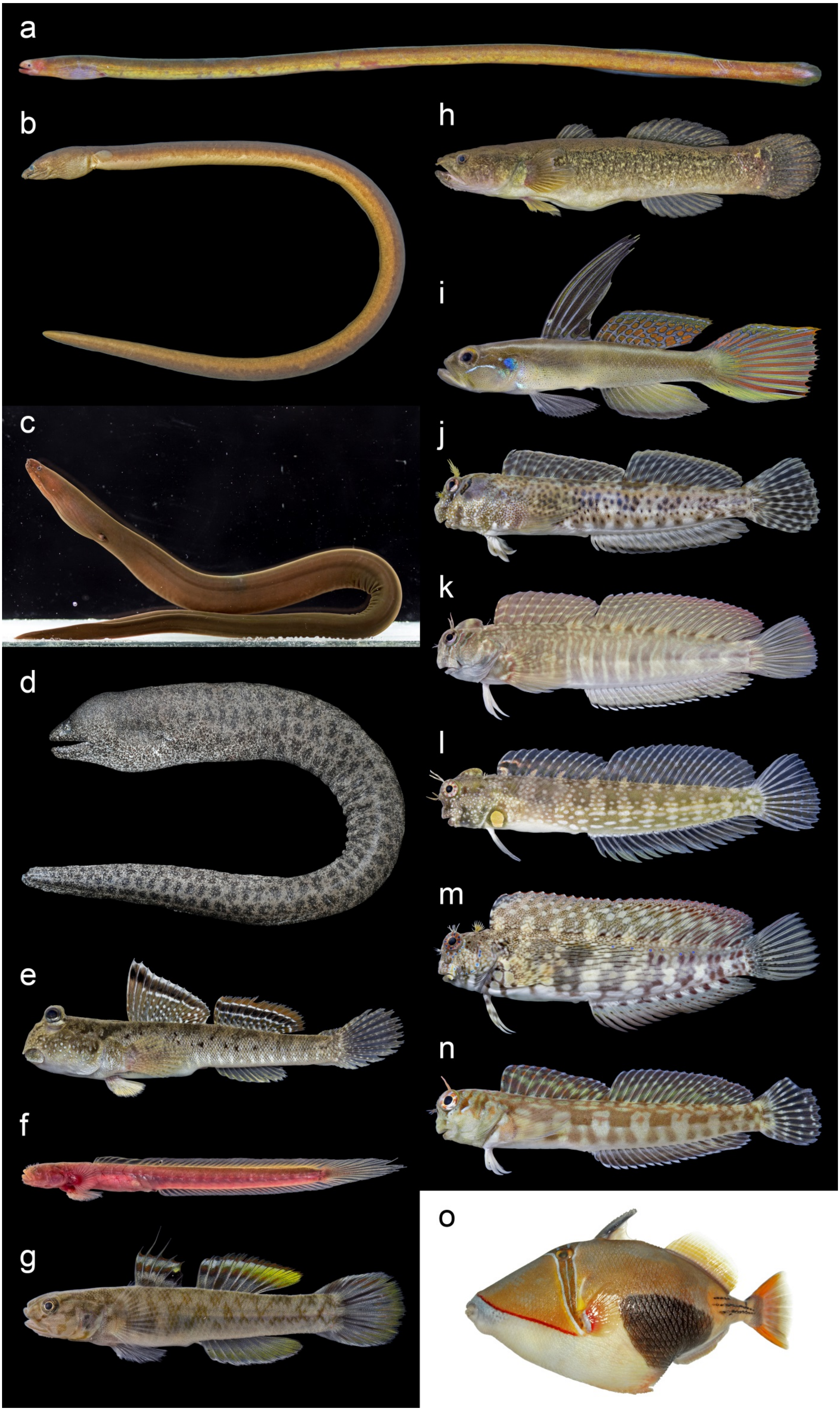
a
b
c
d
e
f
g
h
i
j
k
l
m
n
o
